# Diversity of Cellulolytic Microbes and the Biodegradation of Municipal Solid Waste by a Potential Strain

**DOI:** 10.1155/2012/325907

**Published:** 2012-02-09

**Authors:** S. P. Gautam, P. S. Bundela, A. K. Pandey, Jamaluddin, M. K. Awasthi, S. Sarsaiya

**Affiliations:** ^1^Central Pollution Control Board, New Delhi, India; ^2^Regional office, M. P. Pollution Control Board, Vijay Nagar, Jabalpur, India; ^3^Mycological Research Laboratory, Department of Biological Sciences, Rani Durgavati University, Jabalpur, India; ^4^Yeast and Mycorrhiza Biotechnology Laboratory, Department of Biological Sciences, Rani Durgavati University, Jabalpur, India; ^5^International Institute of Waste Management (IIWM), Bhopal, India

## Abstract

Municipal solid waste contains high amounts of cellulose, which is an ideal organic waste for the growth of most of microorganism as well as composting by potential microbes. In the present study, Congo red test was performed for screening of microorganism, and, after selecting a potential strains, it was further used for biodegradation of organic municipal solid waste. Forty nine out of the 250 different microbes tested (165 belong to fungi and 85 to bacteria) produced cellulase enzyme and among these *Trichoderma viride* was found to be a potential strain in the secondary screening. During the biodegradation of organic waste, after 60 days, the average weight losses were 20.10% in the plates and 33.35% in the piles. There was an increase in pH until 20 days. pH however, stabilized after 30 days in the piles. Temperature also stabilized as the composting process progressed in the piles. The high temperature continued until 30 days of decomposition, after which the temperature dropped to 40°C and below during the maturation. Good quality compost was obtained in 60 days.

## 1. Introduction

In the present technoeconomic era, the energy and environmental crises developed due to huge amount of cellulosic materials are disposed as “waste.” Municipal solid waste is composed of 40–50% cellulose, 9–12% hemicelluloses, and 10–15% lignin on a dry weight basis [1, 2]. Annually, Asia alone generates 4.4 billion tons of solid wastes, and municipal solid waste comprises 790 million tons of which about 48 million tons are generated in India. By the year 2047, municipal solid waste generation in India is expected to reach 300 million tons and land requirement for disposal of this waste would be 169.6 km^2^. Unscientific disposal causes an adverse impact on all components of the environment and human health. Microorganism performs their metabolic processes rapidly and with remarkable specificity under ambient conditions, catalyzed by their diverse enzyme-mediated reactions. An enzyme alternative to harsh chemical technologies has led to intensive exploration of natural microbial biodiversity to discover enzymes. There is a wide spectrum of microorganisms which can produce the variety of enzymes like cellulase under appropriate conditions. 

Cellulases are a consortium of free enzymes comprised of endoglucanases (*β*-1,4-D-glucan-4-glucanohydrolase, EC 3.2.1.4, carboxymethyl cellulase, EC), exoglucanases (*β*-1,4-D-glucan-4-glucohydrolase, EC 3.2.1.91, cellobiohydrolase, CBH), and cellobiases (*β*-D-glucoside glucohydrolase, EC 3.2.1.21, *β*-1,4-D-glucosidase) are found in many of the 57 glycosyl hydrolase families [3]. Several studies were carried out to produce cellulolytic enzymes in organic waste degradation process by several microorganism including fungi such as *Trichoderma* sp., *Penicillium *sp., and *Aspergillus* spp. respectively [4–6]. Many fungi capable of degrading cellulose synthesize large quantities of extracellular cellulases that are more efficient in depolymerising the cellulose substrate. Most commonly studied cellulolytic organisms include fungal species: *Trichoderma*, *Humicola, Penicillium,* and *Aspergillus *[7]. Among *Trichoderma* spp., *T. harzianum* [8–11] and *T. koningii* [12, 13] have been studied. Already an impressive collection of more than 14,000 fungi which were active against cellulose and other insoluble fibres were collected [14]. Many cellulases produced by bacteria appear to be bound to the cell wall and are unable to hydrolyze native lignocellulose preparations to any significant extent. A wide variety of Gram-positive and Gram-negative species are reported to produce cellulose, including *Clostridium thermocellum, Streptomyces *spp*., Ruminococcus *spp.,* Pseudomonas *spp*., Cellulomonas *spp*., Bacillus *spp*., Serratia, Proteus, Staphylococcus *spp*., *and* Bacillus subtilis* [12, 15]. Various biological studies have been carried out to identify the major microbiological agents responsible for biodegradation. Today, environmental policies and regulation progress lead to the development of biodegradation processes to turn organic wastes into a valuable resource by potential microbes because only few strains are capable of secreting a complex of cellulase enzymes, which could have practical application in the enzymatic hydrolysis of cellulose as well as biodegradation of organic municipal solid waste.

Thus, the present work mainly focused on selecting a potential strain and utilization of cellulosic waste for value-added products.

## 2. Materials and Methods

### 2.1. Collection of Samples

**Figure 1:**
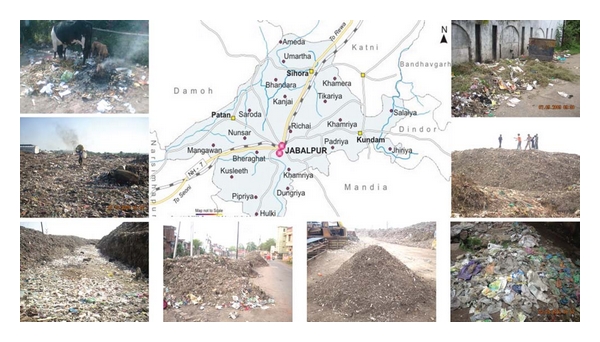
Collection of samples from different waste dump sites of Jabalpur.

Sampling sites were chosen, such that enough cellulose can be accessible naturally, whereby the resident microbial population could predominantly be cellulolytic by nature which could be isolated easily in large numbers. The samples were then brought to the laboratory for microbiological study. Municipal solid waste (MSW), compost, and soil samples collected from different areas of Jabalpur were screened for the isolation of cellulose-degrading microbes. All samples brought to laboratory for isolation were processed within 3–5 h of collection to minimize saprophytic developments (Figure 1).

### 2.2. Isolation, Identification, and Maintenance of Microbial Strains

Samples were collected from a depth of 2 cm and were carried to laboratory in sterilized polythene bags. Soil dilution plate method [16] was employed in the present work to isolate different microbes. The 100 *μ*L portions (10^−4^ & 10^−6^) of the suspensions were inoculated onto plates containing potato dextrose agar (PDA) and nutrient agar media (NAM). The plates were incubated at 30 ± 2°C for 4–8 days. All the isolates obtained from Jabalpur MSW, and composts and soils were identified according to morphological and biochemical basis [17–21]. The identified strains were maintained on PDA and NAM slants at low temperature (4 ± 1°C).

### 2.3. Primary Screening for Cellulolytic Activity

The isolated microbes were grown on basal salt media supplemented with 1% carboxymethylcellulose used for both bacteria and fungi [22]. The pure cultures were inoculated in the centre with almost equal amounts and incubated at 30 ± 2°C until substantial growth was recorded. The Petri plates were flooded with Congo red solution (0.1%), and after 5 min the Congo red solution was discarded, and the plates were washed with 1 M NaCl solution allowed to stand for 15–20 minutes. The clear zone was observed around the colony when the enzyme had utilized the cellulose.

### 2.4. Secondary Screening for Cellulolytic Activity

Potential microbes presenting large clearing zones in Congo red test were used for enzyme production on basal salt medium containing 1% cellulose as a sole carbon source [23]. Shake flask technique was used and 150 mL Erlenmeyer flask filled with 50 mL of the above medium. After autoclaving, each flask was inoculated with two discs (2 mm diameters) cut from the periphery of 6-day-old culture of fungi actively growing on PDA and a loopful of bacterial culture actively growing on NAM. The flasks were then incubated at 30°C in a shaker for 6 days. The flasks were filtered through Whatman number 1 filter paper to separate culture filtrates. The filtrate was analysed for enzyme activities.

### 2.5. Measurement of Enzyme Activity

Filter paper activity (FPase) for total cellulase activity in the culture filtrate was determined according to the standard method. Aliquots of appropriately diluted cultured filtrate as enzyme source were added to a Whatman number 1 filter paper strip (1 × 6 cm; 50 mg) immersed in one milliliter of 0.05 M sodium citrate buffer of pH 4.8. After incubation at 50 ± 2°C for 1 hrs, the reducing sugar released was estimated by dinitrosalicylic acid (DNS) method [24]. One unit of filter paper (FPU) activity was defined as the amount of enzyme-releasing 1 *μ*mole of reducing sugar from filter paper per mL per min. Endoglucanase activity (CMCase) was measured using a reaction mixture containing 1 mL of 1% carboxymethyl cellulose (CMC) in 0.05 M sodium citrate buffer (pH 4.8) and aliquots of suitably diluted filtrate. The reaction mixture was incubated at 50 ± 2°C for 1 h, and the reducing sugar produced was determined by DNS method [24]. *β*-glucosidase activity was assayed by the method of pointing [25]. One unit (IU) of enzyme activity was defined as the amount of enzyme releasing 1 *μ*mole of reducing sugar per min. 

### 2.6. Biodegradation of Municipal Solid Waste

#### 2.6.1. Inoculum Preparation

The fungus used in the study was *Trichoderma viride.* Two discs of fungal mycelium of *T. viride* were subcultured in PDA for mass cultivation and incubated at 30 ± 2°C for 6 days. After 144 hours of growth, 5% (v/v) of spore suspension of *T. viride* culture was mixed with municipal solid waste for bioconversion [26, 27].

#### 2.6.2. Biodegradation of Municipal Solid Waste

Municipal solid wastes were collected from different waste dumping points of Jabalpur in polythene bags. Samples were cut into small pieces, and 5 g of each was aliquoted into petri plates, which were then wrapped by using polythene bags. The plates containing municipal solid waste were then autoclaved at 121°C for 15 min, and 25 kg of waste in polythene bags was also autoclaved for preparing piles. After sterilization, the inoculum was inoculated in a petri plate and piles in triplicate. Moisture content was maintained at 50–60% throughout the active biodegradation in the plates as well as piles. The pH and temperature were also measured periodically in the piles after 10 days intervals. Turning of the organic waste was provided once in every week to ensure aerobic condition both in the piles and plates. Changes in odour and weight loss of the decomposed organic solid waste were observed at 10-day intervals in the piles and at 30 days in the plates up to 60 days. For measurement of weight loss (%), the following formula was used:

(1) $$\text{Weight}\;\text{loss}\;\left(\%\right)=W\frac{W_{1}}{W}\times 100,$$

where *W* is initial weight, and *W*_1_ is final weight.

## 3. Results and Discussion

A total of 250 isolates were isolated; of these, 165 belonged to 37 fungal species, and 85 to 21 bacterial species. These were *Absidia* sp., *Alternaria alternata*, *Alternaria* sp., *Acremonium butyri, Aspergillus clavatus, Aspergillus flavus*, *Aspergillus fumigatus*, *Aspergillus nidulans*, *Aspergillus niger*, *Aspergillus candidus*,* Aspergillus luchuensis*, *Aspergillus terreus*,*   Aspergillus* sp., *Chaetomium* sp., *Chrysosporium* sp., *Cladosporium* sp., *Colletotrichum* sp., *Curvularia lunata*, *Curvularia* sp., *Drechslera* sp., *Exserohilum *sp., *Fusarium oxysporum*, *Fusarium roseum*, *Gliocladium* sp., *Helminthosporium* sp., *Humicola* sp., *Mucor *sp., *Myrothecium* sp., *Penicillium digitatum*, *Penicillium* sp., *Rhizopus* sp., *Sclerotium rolfsii, Torula* sp., *Trichoderma viride*,* Trichoderma *sp., *Verticillium* sp., MRLB #38, MRLB #39, MRLB #40, MRLB #41, MRLB #42, MRLB #43, MRLB #44, MRLB #45, MRLB #46, MRLB #47, MRLB #48, MRLB #49, MRLB #50, MRLB #51, MRLB #52, MRLB #53, MRLB #54, MRLB #55, MRLB #56, MRLB #57, and MRLB #58 (Table 1). The most frequent fungi were *Aspergillus niger*, *Curvularia lunata*, *A. nidulans*, *A. fumigatus, Penicillium* sp., *Fusarium roseum*, and* Trichoderma viride*, and bacteria were MRLB #39, MRLB #42, and MRLB #44. Most of the above isolates have been reported as cellulase producers, but with variable capabilities by several workers [28–33]. Strom [34] reported that a large majority of the total number of bacterial isolates were members of the genus *Bacillus*. Proom and Knight [35] studied the bacteria required minimal nutritional requirement. Ezekiel et al. [36] isolated 22 different cellulolytic fungi from different sites. Duncan et al. [37] also isolated 72 fungi and screened for cellulase activity by using the carboxymethyl cellulose (CMC) Congo red plate technique.

### 3.1. Primary Screening for Cellulolytic Activity

**Figure 2:**
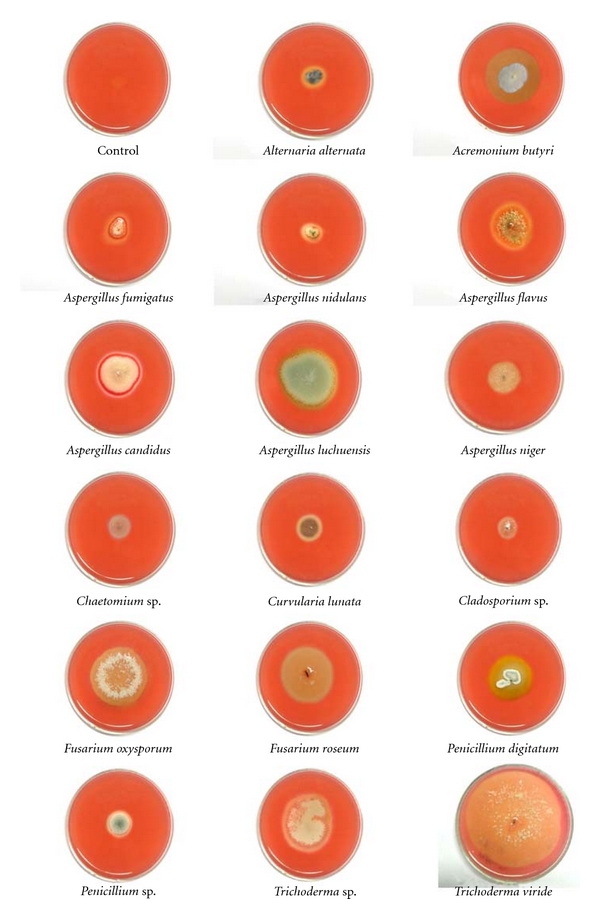
Primary screening of fungi isolated from different sources.

**Figure 3:**
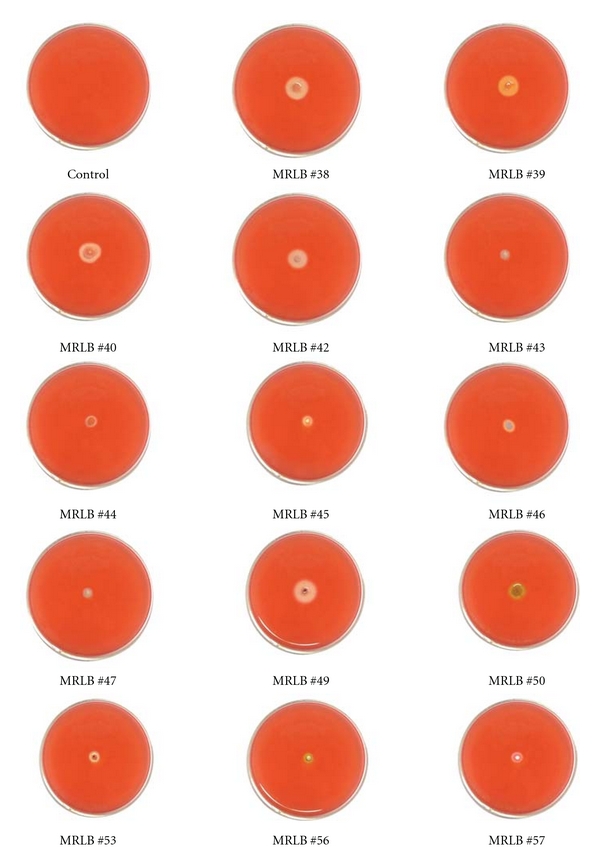
Primary screening of bacteria isolated from different sources.

The results of primary screening showed that degradation of cellulose by tested isolates differs from organism to organism. Out of two hundred fifty isolates tested, cellulolytic activity was detected in only 49 different isolates after 4 days of incubations, indicating them to be cellulose degraders. The diameter of the yellow halo varied from organism to organism. The data present in Table 1 revealed that *Alternaria alternata*, *Alternaria* sp., *Acremonium butyri, Aspergillus clavatus, Aspergillus flavus*, *Aspergillus candidus, Aspergillus luchuensis, Aspergillus fumigatus*, *Aspergillus nidulans*, *Aspergillus niger*, *Aspergillus terreus*, *Aspergillus* sp., *Chaetomium* sp., *Chrysosporium* sp., *Cladosporium* sp., *Curvularia lunata*, *Curvularia* sp., *Drechslera* sp., *Fusarium oxysporum*, *Fusarium roseum, Gliocladium* sp., *Humicola *sp., *Mucor *sp., *Myrothecium* sp., *Paecilomyces* sp., *Penicillium digitatum*, *Penicillium *sp*., Rhizopus* sp., *Sclerotium rolfsii, Trichoderma viride*, *Trichoderma* sp., *Verticillium* sp., MRLB #38, MRLB #39, MRLB #40, MRLB #42, MRLB #43, MRLB #44, MRLB #45, MRLB #46, MRLB #47, MRLB #49, MRLB #50, MRLB #51, MRLB #53, MRLB #54, MRLB #55, MRLB #56, and MRLB #57 were active cellulase producers (Figures 2 and 3). On the other hand, *Absidia* sp., *Colletotrichum* sp., *Exserohilum* sp., *Helminthosporium* sp., *Torula* sp., MRLB #41, MRLB #48, MRLB #52, and MRLB #58 were noncellulose producers. The above species were isolated with different numbers and frequencies from various sources in many places of the world by several workers [38–44]. Of the 250 isolates, 49 different isolates were tested for the production of cellulolytic enzyme (secondary screening).

### 3.2. Secondary Screening for Cellulolytic Activity

It is evident from the results that maximum cellulases activity was observed after the 6th day of incubation at 30°C in *Trichoderma viride*, *Penicillium digitatum*, *Aspergillus niger*, *Chaetomium* sp., and MRLB #38 and MRLB #40 followed by *Aspergillus fumigatus*, *Aspergillus nidulans*, *Alternaria alternata*, *Aspergillus* sp., *Fusarium oxysporum*, *Humicola* sp., MRLB #39, and MRLB #42. Out of the 49 isolates, exo-*β*-glucanase (C_1_) activity in fourteen isolates (*Aspergillus flavus* (0.07 IU/mL)*, Aspergillus terreus* (0.09 IU/mL)*, Aspergillus *sp. (0.10 IU/mL),* Curvularia lunata *(0.11 IU/mL),* Drechslera* sp. (0.15 IU/mL),* Mucor *sp. (0.23 IU/mL), *Rhizopus* sp. (0.13 IU/mL),* Verticillium* sp. (0.20 IU/mL), MRLB #42 (0.08 IU/mL), MRLB #44 (0.16 IU/mL), MRLB #51 (0.08 IU/mL), MRLB #54 (0.14 IU/mL), MRLB #56 (0.09 IU/mL), and MRLB #57 (0.12 IU/mL)) were very low. Maximum exo-*β*-glucanase (C_1_) activity was observed by *Trichoderma viride *(2.22 IU/mL), *Aspergillus niger *(2.05 IU/mL), *Fusarium oxysporum *(1.14 IU/mL), *Fusarium roseum *(1.67 IU/mL), *Penicillium digitatum* (1.38 IU/mL), MRLB #45 (1.66 IU/mL), MRLB #38 (1.65 IU/mL) followed by *Chaetomium* sp. (1.54 IU/mL), *Alternaria alternata* (0.30 IU/mL), *Aspergillus nidulans* (1.19 IU/mL), *Humicola* sp. (1.28 IU/mL), MRLB #39 (1.07 IU/mL), MRLB #47 (1.07 IU/mL), and MRLB #53 (1.34 IU/mL). Maximum endo-*β*-glucanase (C*_x_*) activity was observed in *Trichoderma viride* (2.03 IU/mL), *Aspergillus niger *(1.76 IU/mL),* Aspergillus nidulans *(1.66 IU/mL) and MRLB #38 (1.81 IU/mL) followed by *Chaetomium* sp. (1.19 IU/mL), *Fusarium oxysporum *(1.62 IU/mL), *Fusarium roseum *(1.21 IU/mL), *Humicola *sp. (1.43 IU/mL), MRLB #38 (1.71 IU/mL), MRLB #45 (1.06 IU/mL), MRLB #47 (0.96 IU/mL), and MRLB #53 (1.29 IU/mL). On the other hand, *Alternaria* sp. (0.06 IU/mL), *Aspergillus flavus* (0.04 IU/mL), *Aspergillus terreus* (0.05 IU/mL), *Curvularia lunata* (0.09 IU/mL), *Rhizopus *sp. (0.09 IU/mL), MRLB #42 (0.05 IU/mL), MRLB #51 (0.11 IU/mL), MRLB #56 (0.11 IU/mL), and MRLB #57 (0.16 IU/mL) were showed very low endo-*β*-glucanase activity. *β*-glucosidase activity was detected in all the isolates, reaching to its maximum in *Trichoderma viride *(1.98 IU/mL), *Aspergillus niger* (1.96 IU/mL) and *Pencillium digitatum* (1.53 IU/mL) followed by *Aspergillus nidulans *(1.54 IU/mL), *Chaetomium* sp. (1.34 IU/mL), *Fusarium oxysporum *(1.86 IU/mL), *Fusarium roseum *(1.46 IU/mL), *Humicola* sp. (1.27 IU/mL), *Penicillium* sp. (1.08 IU/mL), MRLB #38 (1.59 IU/mL), MRLB #45 (1.12 IU/mL), and MRLB #53 (1.36 IU/mL). Reference [45] reported *β*-glucosidase production by 39 fungi and found *A. fumigatus* and *A. nidulans* as good *β*-glucosidase producers. The maximum *β*-glucosidase activity reported by [46] was in *Chaetomium cellulotricum*. In the present investigation (Table 2), *Trichoderma viride* was potential strain in the secondary screening and used further for utilization of municipal solid waste for production of value-added product (compost).

### 3.3. Bioconversion of Municipal Solid Waste

**Figure 4:**
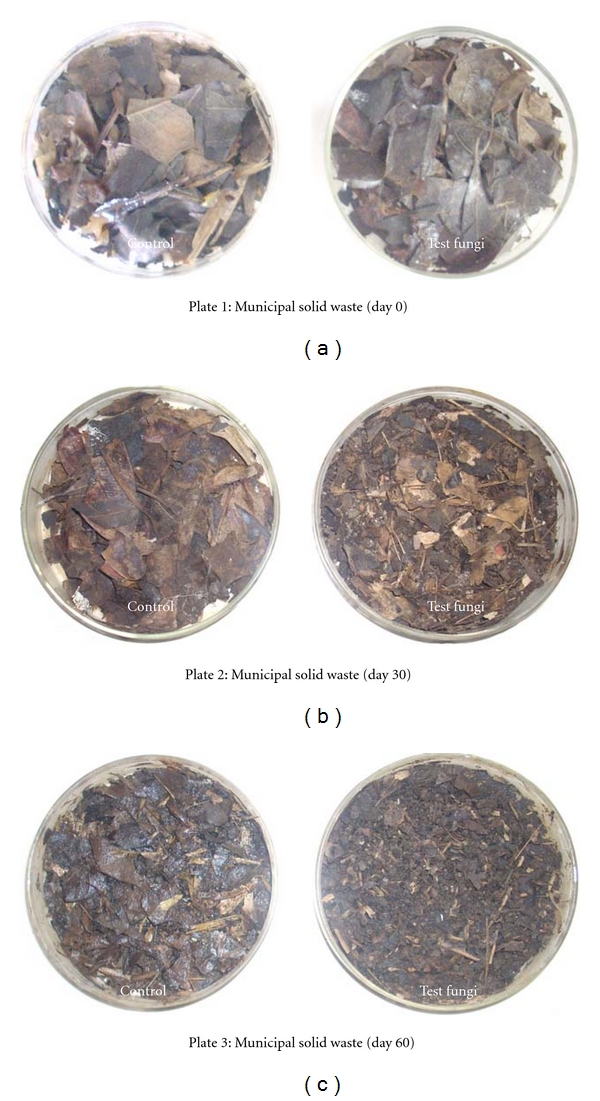
Bioconversion of municipal solid waste by using spore suspension of *T. viride* in petri plates.

**Figure 5:**
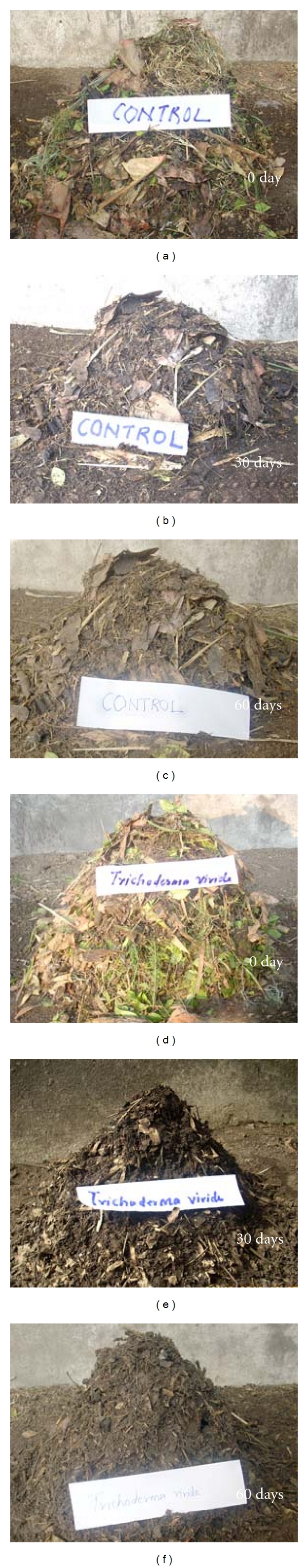
Bioconversion of municipal solid waste by *T. viride *using spore suspension in small piles.

**Figure 6:**
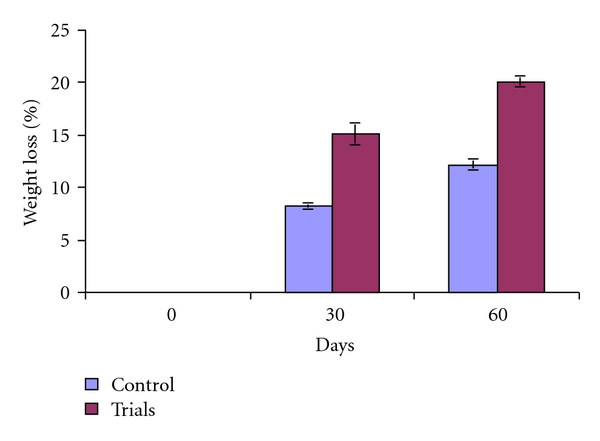
Percent weight loses of municipal solid waste by spore suspension of *T. viride *in plates (5 g).

**Figure 7:**
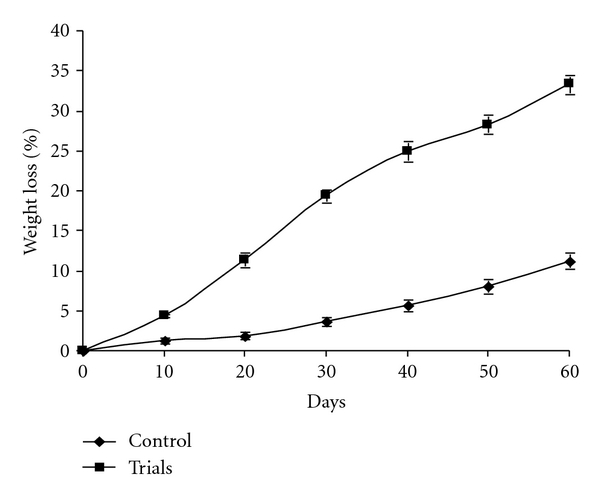
Percent weight loses of municipal solid waste by spore suspension of *T. viride* in open piles (25 Kg).

Fungi play an important role in the decomposition of organic waste and can be important contributors to optimal waste bioconversion. For decomposition of organic solid waste by using spore suspension treatment of *T. viride*, no bad smell was emitted after 60 days. It indicates the possible complete degradation of organic waste in plates that contained 5 g organic waste (Figure 4). In case of the control, the bad smell continued even after 60 days, and it indicates slow degradation. Data presented in the Figure 6 showed that, after 60 days, the average weight loss in three trials (plates) was 12.51% and 20.10%. On the other hand, piles that contained 25 kg of waste in triplicates (Figures 5 and 7) showed that, after 60 days, the average weight loss in three piles was 33.35% and 11.24% in control. 

**Figure 8:**
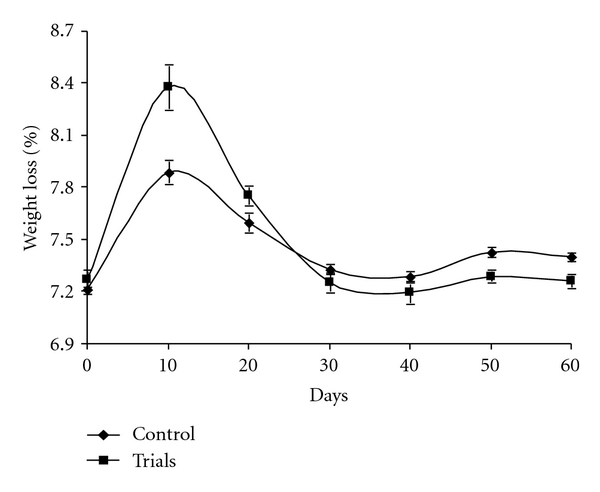
Variation of pH during the bioconversion of MSW.

During the bioconversion of organic waste, there was a shift in pH from the initial condition neutral (7.21 and 7.27) toward an alkaline condition in the piles. The occurrence of these conditions may be attributed to the bioconversion of the organic material into various intermediate types of organic acid and higher mineralization of the nitrogen and phosphorous into nitrites/nitrates and orthophosphate, respectively. This increase in pH during the biodegradation process could be due to the production of ammonium as a result of the ammonification process [47]. Our data show a similar trend (Figure 8) with a fast pH increase during the first ten days of bioconversion and then stabilization with pH fluctuations between 7.33 and 7.25 after 30 days. At the beginning of the experiment, the fluctuations can be explained by the industrial process used, involving periodic turnings and consequently the volatilization of NH_4_^+^. This acid production results from a lack of oxygen that can occur between two turnings. In such conditions, pH can reach values of about 6.0 [48]. Our results show that the pH of composts decreased to a final, mature pH of approximately 7.40 (control) and 7.26, which meets the compost regulations of pH 5.0–8.0 for the US, and pH 5.5–8.0 for the Council of European Communities (CEC) [49].

**Figure 9:**
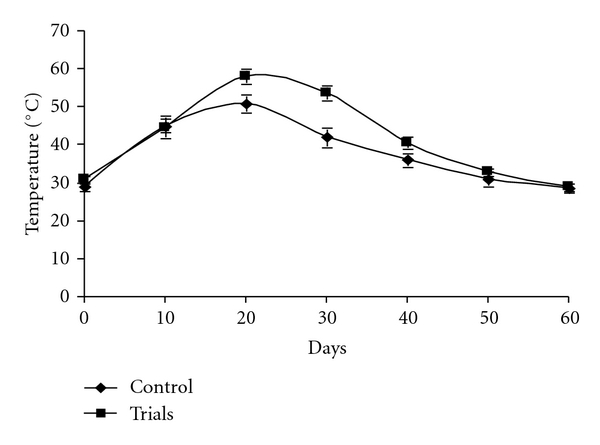
Variation of temperature during the bioconversion of MSW.

The initial average temperature of the turned piles was 29 and 31°C and rapidly rose to a peak of 58°C after 20 days of decomposing. The high temperature continued until 30 days of decomposition, after which the temperature dropped to 36°C by the 40th day of composting. Thereafter, the temperature varied within a narrow range (36–30°C) (Figure 9). The temperature levels in the compost piles tended to increase and reach 40–60°C due to the energy released from the biochemical reactions of the microorganisms in the compost piles, while the temperature levels in the compost piles tended to decrease after the thermophilic phase due to a loss of the substrate and a decrease in microbial activity [50]. It was previously reported that the compost material can be considered mature when an ambient temperature of 28°C is reached [51]. Therefore, this parameter is considered as a good indicator for the end of the biooxidative phase in which the compost achieves some degree of maturity [52–54]. After 60 days, the mature compost was black in color, granular, and fibrous with a pleasant earthy smell. The appearance of black color indicated its maturity. In the case of the control (without culture), the biodegradation was very slow, and weight loss was also low. In order to assess the compost maturity, both the compost samples were placed separately in a sealed polythene bags for a week. After a week, the sealed bags were broken, and the odor was checked, which was found to have earthy smell in the compost produced by *T. viride*, indicating the quality of stable and mature compost and compost produced by without treated *T. viride*, produced bed smell after one week. These findings are in accordance with the previous study where it was reported that mature compost produces an earthy smell after being sealed in the polythene bag for a week [55].

## 4. Conclusions

From the above findings, it can be concluded that a large number of microorganism were found in municipal solid waste, compost, and soil. Municipal solid waste is suitable for composting because of the presence of high percentages of organic matter. The *T. viride* had promising effects in the decomposition of organic municipal solid waste, resulting in a greater bioconversion of the original material than the control. Therefore, pH and temperature were considered as a good indicator for the end of the bioconversion of municipal solid waste in which the compost achieves some degree of maturity.

## Figures and Tables

**Table 1:** Preliminary screening of cellulose-degrading microbes isolated from different sources.

Culture	Code no.	Source			Zone
		Soil	MSW	Compost	
*Absidia* sp.	MRLF #1	+	−	−	−
*Alternaria alternata*	MRLF #2	+	+	+	+
*Alternaria* sp.	MRLF #3	+	+	+	+
*Acremonium butyri*	MRLF #4	+	+	−	+
*Aspergillus clavatus*	MRLF #5	+	+	+	+
*Aspergillus flavus*	MRLF #6	+	+	−	+
*Aspergillus candidus*	MRLF #7	+	−	−	+
*Aspergillus luchuensis*	MRLF #8	+	+	−	+
*Aspergillus fumigatus*	MRLF #9	+	+	+	+
*Aspergillus nidulans*	MRLF #10	+	+	+	+
*Aspergillus niger*	MRLF #11	+	+	+	+
*Aspergillus* sp.	MRLF #12	+	−	−	+
*Aspergillus terreus*	MRLF #13	−	+	−	+
*Chaetomium* sp.	MRLF #14	−	+	−	+
*Chrysosporium* sp.	MRLF #15	+	−	−	+
*Cladosporium* sp.	MRLF #16	+	+	−	+
*Colletotrichum* sp.	MRLF #17	+	+	+	−
*Curvularia lunata*	MRLF #18	+	+	+	+
*Curvularia* sp.	MRLF #19	+	+	+	+
*Drechslera* sp.	MRLF #20	−	+	−	+
*Exserohilum *sp.	MRLF #21	−	+	−	−
*Fusarium oxysporum*	MRLF #22	+	+	+	+
*Fusarium roseum *	MRLF #23	−	+	+	+
*Gliocladium* sp.	MRLF #24	+	−	−	+
*Helminthosporium* sp.	MRLF #25	−	+	−	−
*Humicola* sp.	MRLF #26	+	+	−	+
*Mucor *sp.	MRLF #27	+	+	+	+
*Myrothecium* sp.	MRLF #28	−	+	−	+
*Paecilomyces* sp.	MRLF #29	+	+	−	+
*Penicillium digitatum*	MRLF #30	−	−	+	+
*Penicillium* sp.	MRLF #31	−	+	−	+
*Rhizopus* sp.	MRLF #32	+	+	+	+
*Sclerotium rolfsii*	MRLF #33	−	+	+	+
*Torula* sp.	MRLF #34	−	+	−	−
*Trichoderma viride*	MRLF #35	−	+	+	+
*Trichoderma *sp.	MRLF #36	+	+	−	+
*Verticillium* sp.	MRLB #37	−	+	+	+
Bacteria 1	MRLB #38	−	+	+	+
Bacteria 2	MRLB #39	+	+	+	+
Bacteria 3	MRLB #40	+	+	−	+
Bacteria 4	MRLB #41	+	−	−	−
Bacteria 5	MRLB #42	−	+	+	+
Bacteria 6	MRLB #43	+	+	+	+
Bacteria 7	MRLB #44	+	+	+	+
Bacteria 8	MRLB #45	−	−	+	+
Bacteria 9	MRLB #46	+	+	−	+
Bacteria 10	MRLB #47	+	+	−	+
Bacteria 11	MRLB #48	+	−	+	−
Bacteria 12	MRLB #49	−	−	+	+
Bacteria 13	MRLB #50	+	+	+	+
Bacteria 14	MRLB #51	−	+	+	+
Bacteria 15	MRLB #52	+	+	+	−
Bacteria 16	MRLB #53	+	−	−	+
Bacteria 17	MRLB #54	+	+	−	+
Bacteria 18	MRLB #55	+	+	+	+
Bacteria 19	MRLB #56	+	+	+	+
Bacteria 20	MRLB #57	+	−	+	+
Bacteria 21	MRLB #58	+	+	+	−

−: Absent; +: present; MSW: municipal solid waste.

**Table 2:** Secondary screening of cellulose-producing microorganism.

Culture	Code no.	Enzyme activity (IU/mL)		
		Exoglucanase	Endoglucanase	**β**-glucosidase
*Alternaria alternata*	MRLF #2	0.30 ± 0.02	0.58 ± 0.03	0.16 ± 0.01
*Alternaria* sp.	MRLF #3	0.19 ± 0.01	0.06 ± 0.007	0.12 ± 0.01
*Acremonium butyri*	MRLF #4	0.32 ± 0.01	0.45 ± 0.02	0.29 ± 0.01
*Aspergillus clavatus*	MRLF #5	0.46 ± 0.02	0.52 ± 0.03	0.40 ± 0.03
*Aspergillus flavus*	MRLF #6	0.07 ± 0.003	0.04 ± 0.005	0.10 ± 0.008
*Aspergilluscandidus*	MRLF #7	0.43 ± 0.02	0.36 ± 0.01	0.50 ± 0.02
*Aspergillus luchuensis*	MRLF #8	0.65 ± 0.02	0.71 ± 0.04	0.53 ± 0.04
*Aspergillus fumigatus*	MRLF #9	0.72 ± 0.03	0.58 ± 0.03	0.85 ± 0.05
*Aspergillus nidulans*	MRLF #10	1.19 ± 0.05	1.66 ± 0.06	1.54 ± 0.06
*Aspergillus niger*	MRLF #11	2.05 ± 0.06	1.76 ± 0.06	1.96 ± 0.06
*Aspergillus terreus*	MRLF #12	0.09 ± 0.005	0.05 ± 0.006	0.08 ± 0.005
*Aspergillus* sp.	MRLF #13	0.10 ± 0.007	0.14 ± 0.01	0.13 ± 0.01
*Chaetomium* sp.	MRLF #14	1.54 ± 0.04	1.19 ± 0.05	1.34 ± 0.05
*Chrysosporium* sp.	MRLF #15	0.29 ± 0.01	0.37 ± 0.02	0.26 ± 0.01
*Cladosporium* sp.	MRLF #16	0.82 ± 0.05	0.91 ± 0.05	0.65 ± 0.04
*Curvularia lunata*	MRLF #18	0.11 ± 0.01	0.09 ± 0.005	0.15 ± 0.01
*Curvularia* sp.	MRLF #19	0.32 ± 0.06	0.39 ± 0.04	0.27 ± 0.01
*Drechslera* sp.	MRLF #20	0.15 ± 0.04	0.17 ± 0.01	0.21 ± 0.02
*Fusarium oxysporum*	MRLF #22	1.14 ± 0.07	1.62 ± 0.06	1.86 ± 0.06
*Fusarium roseum *	MRLF #23	1.67 ± 0.06	1.21 ± 0.04	1.46 ± 0.05
*Gliocladium* sp.	MRLF #24	0.61 ± 0.03	0.78 ± 0.04	0.59 ± 0.03
*Humicola *sp.	MRLF #26	1.28 ± 0.04	1.43 ± 0.05	1.27 ± 0.06
*Mucor *sp.	MRLF #27	0.23 ± 0.01	0.41 ± 0.02	0.32 ± 0.02
*Myrothecium* sp.	MRLF #28	1.21 ± 0.05	0.99 ± 0.03	0.91 ± 0.04
*Paecilomyces* sp.	MRLF #29	0.51 ± 0.02	0.84 ± 0.03	0.64 ± 0.03
*Penicillium digitatum*	MRLF #30	1.38 ± 0.06	1.37 ± 0.05	1.53 ± 0.06
*Penicillium* sp.	MRLF #31	0.91 ± 0.03	0.72 ± 0.03	1.08 ± 0.04
*Rhizopus* sp.	MRLF #32	0.13 ± 0.009	0.09 ± 0.006	0.14 ± 0.007
*Sclerotium rolfsii*	MRLF #33	0.67 ± 0.03	0.71 ± 0.05	0.68 ± 0.04
*Trichodermaviride*	MRLF #35	2.22 ± 0.07	2.03 ± 0.06	1.98 ± 0.06
*Trichoderma* sp.	MRLF #36	0.76 ± 0.04	0.78 ± 0.04	0.69 ± 0.03
*Verticillium* sp.	MRLF #37	0.20 ± 0.02	0.17 ± 0.01	0.27 ± 0.01
Bacteria 1	MRLB #38	1.65 ± 0.06	1.71 ± 0.06	1.59 ± 0.05
Bacteria 2	MRLB #39	1.07 ± 0.05	0.96 ± 0.04	0.94 ± 0.04
Bacteria 3	MRLB #40	0.73 ± 0.04	0.67 ± 0.03	0.52 ± 0.03
Bacteria 5	MRLB #42	0.08 ± 0.006	0.05 ± 0.003	0.11 ± 0.006
Bacteria 6	MRLB #43	0.32 ± 0.02	0.24 ± 0.01	0.29 ± 0.01
Bacteria 7	MRLB #44	0.16 ± 0.01	0.19 ± 0.009	0.14 ± 0.008
Bacteria 8	MRLB #45	1.66 ± 0.04	1.06 ± 0.04	1.12 ± 0.06
Bacteria 9	MRLB #46	0.76 ± 0.04	0.61 ± 0.02	0.68 ± 0.04
Bacteria 10	MRLB #47	1.07 ± 0.06	0.96 ± 0.03	0.89 ± 0.05
Bacteria 12	MRLB #49	0.21 ± 0.02	0.30 ± 0.02	0.27 ± 0.01
Bacteria 13	MRLB #50	0.64 ± 0.03	0.72 ± 0.04	0.35 ± 0.02
Bacteria 14	MRLB #51	0.08 ± 0.007	0.11 ± 0.009	0.13 ± 0.008
Bacteria 16	MRLB #53	1.34 ± 0.06	1.29 ± 0.05	1.36 ± 0.04
Bacteria 17	MRLB #54	0.14 ± 0.01	0.20 ± 0.01	0.18 ± 0.009
Bacteria 18	MRLB #55	0.28 ± 0.02	0.16 ± 0.01	0.19 ± 0.007
Bacteria 19	MRLB #56	0.09 ± 0.007	0.11 ± 0.01	0.13 ± 0.01
Bacteria 20	MRLB #57	0.12 ± 0.009	0.16 ± 0.01	0.14 ± 0.004

Values are means ± SEm of the three observations.
